# Supplementary material for: Comparative roadmaps of reprogramming and oncogenic transformation identify Bcl11b and Atoh8 as broad regulators of cellular plasticity
Source: Nat Cell Biol. 2022 Sep 8;24(9):1350–63. doi: 10.1038/s41556-022-00986-w (PMC9481462; doi:10.1038/s41556-022-00986-w)
Supplement: Supplementary file 1 — Supplementary Tables 1 and 2. [file 41556_2022_986_MOESM1_ESM.pdf]

nature portfolio
https://doi.org/10.1038/s41556-022-00986-w

Supplementary information

Comparative roadmaps of reprogramming and oncogenic transformation identify Bcl11b and Atoh8 as broad regulators of cellular plasticity

In the format provided by the authors and unedited

Supplementary table 1

List of 150 genes enriched in MEF clusters
Hoxc8
Hoxb9
Loxl2
En1
Akap12
Lrrc15
Hoxc6
Grem2
Ncam1
Galnt17
Gng11
Thy1
Ctla2a
Hoxc9
Nnmt
Rian
F2r
Tmsb10
Nectin2
Hoxc10
Meg3
Flt1
Hoxb2
Ccn4
Thbs2
Tnc
Siglecg
Crispld2
Galnt18
Adam23
Tfpi
Cald1
Rbpms
Ankrd1
Zdbf2
Hoxb8
Gm53
Pde8b
Ptn
Hoxb4
Fibin
Lasp1
Khdrbs3
AW551984
Hoxb7
Mcam

Meis1
Arhgap31
Twist2
Id4
Hoxb3
Ptprz1
Slit3
B830012L14Rik
Tubb2b
C1qtnf3
Chst2
Klk8
Tuba1a
Mn1
Mirg
Tagln
Lhfp
Lmod1
Rpl39l
Ndn
Cdh3
Actg2
Grb10
Cd44
Gm2115
Sh3glb1
Basp1
Peg3
Fyn
Tbxa2r
Meis2
Irx5
S100a11
Kif1a
Dpysl3
Gpr149
Cntfr
Axl
Capn2
Cnrip1
Ch25h
Rbms3
Acta2
Clca3a1
Igfbp7
Enho
Tubb3
Pgm5

Wnt4
Syt13
Mxra7
Rgs12
Tfpi2
F3
Gask1b
Dbn1
Zyx
Cdh4
Fhl2
Kcnab1
Sorcs2
Nid1
Hoxc4
Lyn
Irx1
Tead1
Sptbn1
Adam33
Flna
Anxa6
Rbp1
Wwc2
Esyt1
Bcl11b
Hoxb5os
Pakap
Serpinb9b
Tlcd2
Loxl3
Gpx3
4833412C05Rik
Selenof
Fermt2
Itprip
Lpp
Hspb1
Ptgis
Sc5d
Iglon5
Tgfb1i1
Serpinb6a
Gabrb1
Vcan
Tsc22d2
Thbs1
Dok5

Hoxb5
Tshz2
Hoxb6
Itga4
Mdk
Hoxc5
Palld

Supplementary table 2

Genotyping primers

Transgene	Primer 1 (5'-3')	Primer 2 (5'-3')	Primer 3 (5'-3')
Col1a1^{4F2A}	CCCTCCATGTGTGACCAAGG	TTGCTCAGCGGTGCTGTCCA	GCACAGCATTGCGGACATG
R26^{rtTA}	GCGAAGAGTTTGTCCTCAACC	AAAGTCGCTCTGAGTTGTTAT	GGAGCGGGAGAAATGGATATG
LSL-K-ras^{G12D}	CCTTTACAAGCGCACGCAGACTGTAGA	AGCTAGCCACCATGGCTTGAGTAAGTCTGCA	
R26-CRE^{ERT2}	TGCCACGACCAAGTGACAGC	CCAGGTTACGGATATAGTTCATG	
OCT4-EGFP	CAAGGCAAGGGAGGTAGACA	TGCCAGACAATGGCTATGAG	CCAAAAGACGGCAATATGGT
Bcl11b-tdTomato	GCCGGGTACCGAAGACACCAAACCGCTCTT	GCCCGGATCCTCCCCTTGGCAACTACTGAC	

shRNA sequences (5'-3')

Gene	Sequence
p53	CCCACTACAAGTACATGTGTAA
FosL1	CCAGTGCCTTGCATCTCCCTT
Atoh8	CGTCAATTTCACACGTAATTT
shSfrp1	ACTGGCCCGAGATGCTCAAAT
shBcl11b	CAAGTTCCAGAGCAATCTCAT

qPCR primers (5'-3')

Gene	Forward primer	Reverse primer
Gapdh	CATGGCCTTCCGTGTTCCTA	GCCTGCTTCACCACCTTCTT
Rplp0	GCTGATCATCCAGCAGGTGT	GGACACCCTCCAGAAAGCGA
Bcl11b	GGGAACTCATCACGCCAGAG	TGAGTAGATCAGGGTCGGGG
Atoh8	CCTCAGCTTCTCCGAGTGTG	CAGGTCACTCCTTCCGTTTCT
Gata4	TGGAAGACACCCCAATCTCG	TAGTCTGGCAGTTGGCACAG
Sox17	GACTCCGGTGTGAATCTCCC	TAACACTGCTTCTGGCCTGC
Brachyury (T)	CGCTGTGACTGCCTACCAGAATG	GAGAGAGAGCGAGCCTCCAAAC
Eomes	AGCCATGTTTGCCCTAGTCC	GCTTGCTCTCTCCTGAGTCC
Sfrp1	GGAAGCCTCTAAGCCCCAAG	CATCCTCAGTGCAAACTCGC

Guide CRISPR sequences (5'-3')

	Forward sequence
Atoh8-1	GGAAGCACATCCCGGTCCTCG
Atoh8-2	GCCGGGATGTGCTTCATGGCG

Western blot and FACS antibodies

	Reference	Source	Dilution
BCL11B	ab18465	Abcam	1/1000
b-ACTIN HRP	A3854	Sigma	1/10 000
ATOH8	PA5-20710	Invitrogen	1/1000
ID4	BCH-9/82-12	BioCheck	1/1000
TWIST2	HOO7581-M01	Abnova	1/1000
GAPDH	sc-25778	Santa-Cruz	1/1000
CDH1	610181	BD	1/1000
SNAIL	3895S	Cell signaling	1/1000
VIM	R28	Cell signaling	1/1000
TWIST1	ab50887	Abcam	1/1000
SOX2	ab97959	Abcam	1/1000
SSEA1	sc-101462	Santa-cruz	1/1000
NANOG	RCAB002P	Reprocell	1/1000
OCT4	sc-5279	Santa Cruz Biotechnology	1/1000
CMYC	sc-42	Santa Cruz Biotechnology	1/1000
FOSL1	sc376148	Santa Cruz Biotechnology	1/1000
b-TUBULIN	T5293	Sigma Aldrich	1/1000
AM-tag	91111	Active Motif	1/1000
ACTIVE β-CATENIN	8E7	Millipore	1/1000
SFRP1	ab267466	Abcam	1/1000
KRAS-G12D	8955	Cell signaling	1/1000
hSOX2	AF2018	R&D	1/1000
hNANOG	3580	Cell signaling	1/1000
β-CATENIN	sc-7963	Santa Cruz Biotechnology	1/1000
goat anti-rabbit HRP	111-035-144	Jackson Immuno Reasearch	1/5000
goat anti-mouse HRP	115-035-044	Jackson Immuno Reasearch	1/5000
goat anti-rat HRP	112-035-143	Jackson Immuno Reasearch	1/5000
APC Rat anti-Mouse CD90.2 (Thy-1.2)	17090283	Invitrogen	1/5000

Immunofluorescence antibodies

	Reference	Source	Dilution
NANOG	RCAB001P	Reprocell	1/100

SSEA1	sc-101462	Santa Cruz Biotechnology	1/100
OCT4	sc-5279	Santa Cruz Biotechnology	1/100
SOX2	ab97959	Abcam	1/100
MAP2	M4403	Sigma Aldrich	1/100
phospho-Histone H2A.X	2577	Cell Signaling Technology	1/100
Live SSEA4	SC023B	R&D	1/100
AF555 Donkey anti-Mouse	A31570	Invitrogen	1/500
AF488 Donkey anti-Mouse	A21202	Invitrogen	1/500
AF555 Donkey anti-Rabbit	A31572	Invitrogen	1/500
AF647 Donkey anti-Rabbit	A31573	Invitrogen	1/500

ChIP antibodies

	**Reference**	**Source**
AM-tag	91111	Active motif
BCL11B	ab18465	Abcam
BCL11B	A300-385A	Bethyl Laboratories
