# Supplementary material for: Comparative roadmaps of reprogramming and oncogenic transformation identify Bcl11b and Atoh8 as broad regulators of cellular plasticity
Source: Nat Cell Biol. 2022 Sep 8;24(9):1350–63. doi: 10.1038/s41556-022-00986-w (PMC9481462; doi:10.1038/s41556-022-00986-w)
Supplement: Supplementary file 2 — Reporting Summary [file 41556_2022_986_MOESM2_ESM.pdf]

nature portfolio

Corresponding author(s): Lavial Fabrice

Last updated by author(s): May 6, 2022

Reporting Summary

Nature Portfolio wishes to improve the reproducibility of the work that we publish. This form provides structure for consistency and transparency in reporting. For further information on Nature Portfolio policies, see our Editorial Policies and the Editorial Policy Checklist.

Statistics

For all statistical analyses, confirm that the following items are present in the figure legend, table legend, main text, or Methods section.

n/a	Confirmed	
☐	☒	The exact sample size (*n*) for each experimental group/condition, given as a discrete number and unit of measurement
☐	☒	A statement on whether measurements were taken from distinct samples or whether the same sample was measured repeatedly
☐	☒	The statistical test(s) used AND whether they are one- or two-sided<br>*Only common tests should be described solely by name; describe more complex techniques in the Methods section.*
☒	☐	A description of all covariates tested
☒	☐	A description of any assumptions or corrections, such as tests of normality and adjustment for multiple comparisons
☐	☒	A full description of the statistical parameters including central tendency (e.g. means) or other basic estimates (e.g. regression coefficient) AND variation (e.g. standard deviation) or associated estimates of uncertainty (e.g. confidence intervals)
☐	☒	For null hypothesis testing, the test statistic (e.g. *F*, *t*, *r*) with confidence intervals, effect sizes, degrees of freedom and *P* value noted<br>*Give P values as exact values whenever suitable.*
☒	☐	For Bayesian analysis, information on the choice of priors and Markov chain Monte Carlo settings
☐	☒	For hierarchical and complex designs, identification of the appropriate level for tests and full reporting of outcomes
☒	☐	Estimates of effect sizes (e.g. Cohen's *d*, Pearson's *r*), indicating how they were calculated

Our web collection on statistics for biologists contains articles on many of the points above.

Software and code

Policy information about availability of computer code

Data collection	Western blot acquisition was performed using a Image Lab 6.0.1 Bio-Rad software. Q-RTPCR data were acquired using the Lightcycler 4.1 software. Microscopy images were acquired with axiovision 4.8.2 software. FACS data were acquired on a FACSDiva v8.0 software.
Data analysis	All the analyses conducted are described in the methods section. Quantification of western blot data was performed using ImageJ 1.53r. Most of the data analysis and statistics were performed using Graphpad prism 8. Next-generation sequencing data were analysed using FASTQC (v0.11.5), STAR (v2.5.2b), DESeq2 (v1.30.1) package in R (v4.0.3), GSVA R-package v1.44.0, Cellranger Single-Cell Software Suite (v3.0.2), Cerebro visualization tool v1.3, Bowtie 2.1.0, MACS 2.1.1, seqMINER v1.3.4., MEME Suite v5.4.1, IGV genome browser (v2.4.15), ChromHMM v1.14.

For manuscripts utilizing custom algorithms or software that are central to the research but not yet described in published literature, software must be made available to editors and reviewers. We strongly encourage code deposition in a community repository (e.g. GitHub). See the Nature Portfolio guidelines for submitting code & software for further information.

Data

Policy information about availability of data

All manuscripts must include a data availability statement. This statement should provide the following information, where applicable:

- Accession codes, unique identifiers, or web links for publicly available datasets
- A description of any restrictions on data availability
- For clinical datasets or third party data, please ensure that the statement adheres to our policy

Data availability
NGS data were deposited on GEO (record number series GSE137050).

Sequencing data that support the findings of this study have been deposited in the Gene Expression Omnibus (GEO) under accession code GSE137050. Previously published data that were re-analysed here are available under accession code GSE90895, GSE10871, GSE11074, GSE122662, GSE62777, SRA SRP046744 and SRP119979. Source data are provided with this study. All other data supporting the findings of this study are available from the corresponding author on reasonable request.

Field-specific reporting

Please select the one below that is the best fit for your research. If you are not sure, read the appropriate sections before making your selection.

☒ Life sciences ☐ Behavioural & social sciences ☐ Ecological, evolutionary & environmental sciences

For a reference copy of the document with all sections, see nature.com/documents/nr-reporting-summary-flat.pdf

Life sciences study design

All studies must disclose on these points even when the disclosure is negative.

Sample size	Sample sizes were determined without statistical measures, but based on prior experience with the specific experiments and widely used sizes in relevant publications (Polo. et al., 2012, Ozmadenci D. et al., 2015) within this field of research in order to ensure that it will be appropriate for statistical analysis. See Figures legends for each experiment.
Data exclusions	No data exclusion was used in the study.
Replication	The vast majority of the presented results come from three independent biological replicates and some results from two independent biological replicates. Next-generation-sequencing data are coming from 2 or 3 independent biological replicates. In all cases, all attempts at replication were successful.
Randomization	No randomization was used in the study.
Blinding	Experiments execution, data collection and result analysis were carried out by the same researcher, therefore no blinding was used.

Reporting for specific materials, systems and methods

We require information from authors about some types of materials, experimental systems and methods used in many studies. Here, indicate whether each material, system or method listed is relevant to your study. If you are not sure if a list item applies to your research, read the appropriate section before selecting a response.

Materials & experimental systems

n/a	Involved in the study	
☐	☒	Antibodies
☐	☒	Eukaryotic cell lines
☒	☐	Palaeontology and archaeology
☐	☒	Animals and other organisms
☒	☐	Human research participants
☒	☐	Clinical data
☒	☐	Dual use research of concern

Methods

n/a	Involved in the study	
☐	☒	ChIP-seq
☐	☒	Flow cytometry
☒	☐	MRI-based neuroimaging

Antibodies

Antibodies used	The following antibodies were used: Rat anti-mouse Thy1 (17090283, Invitrogen, 1:1000), mouse anti-OCT4 (sc-5279, Santa Cruz, 1:1000), rabbit anti-SOX2 (ab97959, Abcam, 1:1000, mouse anti-c-MYC (sc-42, Santa-Cruz, 1:200), rabbit-anti ATOH8 (PA5-20710, Termofisher, 1:1000), rabbit anti-ID4 (BCH-9/82-12, BioCheck, 1:1000), MAP2 (Sigma Aldrich, M4403), rabbit anti-NANOG (RCAB002P, Reprocell, 1:1000), mouse anti-SSEA1 (sc-101462, Santa Cruz, 1:1000), mouse AM-Tag (active motif 9111), mouse anti-CDH1 (610181, BD, 1:1000), rabbit anti-SNAIL (3895S, Cell signaling, 1:1000), rabbit anti-VIM (R28, Cell signaling, 1:1000), mouse anti-TWIST1 (ab50887, Abcam, 1:250), goat anti-hSOX2 (AF2018, R&D, 1:1000), rabbit anti-hNANOG (3580, Cell signaling, 1:1000), mouse anti-ACTIVE β-CATENIN (8E7, Millipore, 1:1000), rabbit anti-GAPDH (sc-25778, Santa-Cruz, 1:4000),rat anti-BCL11B (Abcam, ab18465, 1:500), horse radish peroxidase (HRP)-conjugated anti-ACTIN (Sigma Aldrich, A3854, 1:10,000), anti-TWIST2 (Abnova H007581-MO1, 1:1000), anti-FOSL1 (Santa Cruz sc376148, 1:1000), anti-b-Tubulin (Sigma T5293, 1:1000), anti-Sfrp1 (Abcam ab267466, 1:1000), anti-K-RasG12D (Cell signaling 8955, 1:1000), anti-Bcl11b (Bethyl A300-385A), phospho-Histone H2A.X (Cell Signaling Technology 2577). Live SSEA4 immunostaining was carried out with the GloLIVE Human Pluripotent Stem Cell Live Cell Imaging Kit (SC023B, R&D).
Validation	The information of all antibodies we used can be found in their official website.

Eukaryotic cell lines

Policy information about cell lines

Cell line source(s)	All the cell lines sources can be found in the methods. Primary MEFs were derived from E13.5 embryos in the Lavial lab, as previously described (Ozmadenci D. et al., 2015). MAEFs were derived from adult mice at 6-8 weeks old. Mouse iPS cells were derived from MEFs following exposure to OSKM expression (dox-inducible or retroviral, as indicated). Transformed cells (TC) were generated by the serial passaging of MEFs induced to express the indicated oncogenes. Human dermal fibroblasts were pruchased from Sigma (Merck 106-05A). Primary T cells were derived from the spleen and lymph nodes of adult mice (6-8 weeks old). 293T cells were purchased from ATCC (CRL-1573) and Plat-E cells from Cellbiolabs (RV-101).
Authentication	The differentiation potential of the mouse iPS cell lines was authenticated by performing in vivo differentiation in teratoma assays. The differentiation ability of human iPS cell lines was evaluated in vitro by embryoid body formation assay. The malignant fetaures of TC cells was evaluated in vitro by soft agar assays and in vivo by conducting mouse xenograft and chick chorioallantoic membrane assays. The other cell lines were not authenticated.
Mycoplasma contamination	All cells are tested negative for mycoplasma contamination using MycoAlert Mycoplasma detection kit from Lonza.
Commonly misidentified lines (See ICLAC register)	None of the cell lines used in this study is listed in the database of commonly misidentified cell lines maintained by ICLAC.

Animals and other organisms

Policy information about studies involving animals; ARRIVE guidelines recommended for reporting animal research

Laboratory animals	Laboratory animals used in the study are described in the methods. MEFs were derived from E13.5 embryos of repro-transformable C57Bl mice models. Pou5f1-GFP C57Bl mice were also used. Teratoma assays were performed with 7-week-old severe combined immunodeficient (SCID) female mice (CB17/SCID, Charles River). Xenograft assays were performed with 7-week old immunocompromised SCID mice. CAM assays were conducted with E11 chicken embryos (local supplier élevage avicole du grand buisson).
Wild animals	The study did not involve wild animals.
Field-collected samples	The study did not involve samples collected from the field.
Ethics oversight	Animals were maintained in a specific pathogen free (SPF) animal facility P-PAC at the Cancer Research Center of Lyon (CRCL) at the Center Léon Bérard (CLB), Lyon, France. All the experiments were performed in accordance with the animal care guidelines of the European Union and were validated by the local Animal Ethic Evaluation Committee (C2EA 15 agreed by the French Ministery of High Scool and Research).

Note that full information on the approval of the study protocol must also be provided in the manuscript.

ChIP-seq

Data deposition

☒ Confirm that both raw and final processed data have been deposited in a public database such as GEO.

☒ Confirm that you have deposited or provided access to graph files (e.g. BED files) for the called peaks.

Data access links *May remain private before publication.*	Record number series GSE137050, secure token for reviewers access yvmpscquvhybzgl.
Files in database submission	Raw and processed data files:<br>Input-Atoh8-rep1<br>AM-empty-rep1<br>AM-Atoh8-rep1<br>Input-Atoh8-rep2<br>AM-empty-rep2<br>AM-Atoh8-rep2<br>Input-Bcl11b-rep1<br>Bcl11b-rep1<br>Bcl11b-rep2
Genome browser session (e.g. UCSC)	No

Methodology

Replicates	For Atoh8 ChIP-seq, we conducted 2 independent experiments. Each of them includes one input file, one file of MEFs infected with an empty-AM-Tag vector and one file of MEFs infected with an Atoh8-AM-tag vector.<br>For Bcl11b, we conducted two independent ChIP-seq experiments. We provide one input file and 2 Bcl11b-chip-seq files.

Sequencing depth	Sequencing depth per sample:<br>-Input: 38,668,812 sequenced reads<br>-AM-rep1: 27,773,977 sequenced reads<br>-AM-Atoh8-rep1: 34,110,739 sequenced reads<br>-AM-Atoh8 rep2: 40,109,516 sequenced reads<br>-AM-rep2: 34,110,739 sequenced reads<br>-Input_for_Atoh8_rep2: 38,001,912 sequenced reads<br><br>-Bcl11b_rep1: 38,397,339 sequenced reads<br>-Bcl11b_rep2: 32,917,302 sequenced reads<br>-Input_for_Bcl11b: 38,237,984 sequenced reads
Antibodies	mouse AM-Tag (active motif 9111), Bcl11b (Abcam ab18465), Bcl11b (Bethyl A300-385A)
Peak calling parameters	Peak caller MACS2 (version 2.2.7.1), following by default parameters, including the following options: --nomodel, --extsize 150, --control Input_dataset.
Data quality	Excellent. Quality assessment has been performed with the NGS-QC Generator tool (Mendoza-Parra et al. NAR 2013).
Software	Bowtie2 (alignement); MACS2 (peak calling); bedtools (peaks comparison); ChromHMM (peaks comparison with public datasets).

Flow Cytometry

Plots

Confirm that:

☒ The axis labels state the marker and fluorochrome used (e.g. CD4-FITC).

☒ The axis scales are clearly visible. Include numbers along axes only for bottom left plot of group (a 'group' is an analysis of identical markers).

☒ All plots are contour plots with outliers or pseudocolor plots.

☒ A numerical value for number of cells or percentage (with statistics) is provided.

Methodology

Sample preparation	MEF cells were washed with PBS, trypsinized and resuspended in PBS with 5% FBS for FACS.
Instrument	Flow cytometry analysis was performed using the BD Fortessa and sorting was performed on the BD FACSAria II sorter.
Software	BD FACSDiva (v8.0) was used for data collection and FlowJo (v10) was used for data analysis.
Cell population abundance	A total of at least 10,000 events were quantified.
Gating strategy	Stringent gatings were always used, leaving a significant gap in between negative/postive population. Cells sorted by FACS were reanalyzed with BD Fortessa to confirm the purity. A detailed gating strategy can be found in Extended Data Fig. 3c.

☒ Tick this box to confirm that a figure exemplifying the gating strategy is provided in the Supplementary Information.
