# Supplementary figures and images for: Comparative roadmaps of reprogramming and oncogenic transformation identify Bcl11b and Atoh8 as broad regulators of cellular plasticity
Source: Nat Cell Biol. 2022 Sep 8;24(9):1350–63. doi: 10.1038/s41556-022-00986-w (PMC9481462; doi:10.1038/s41556-022-00986-w)

Figure 2d

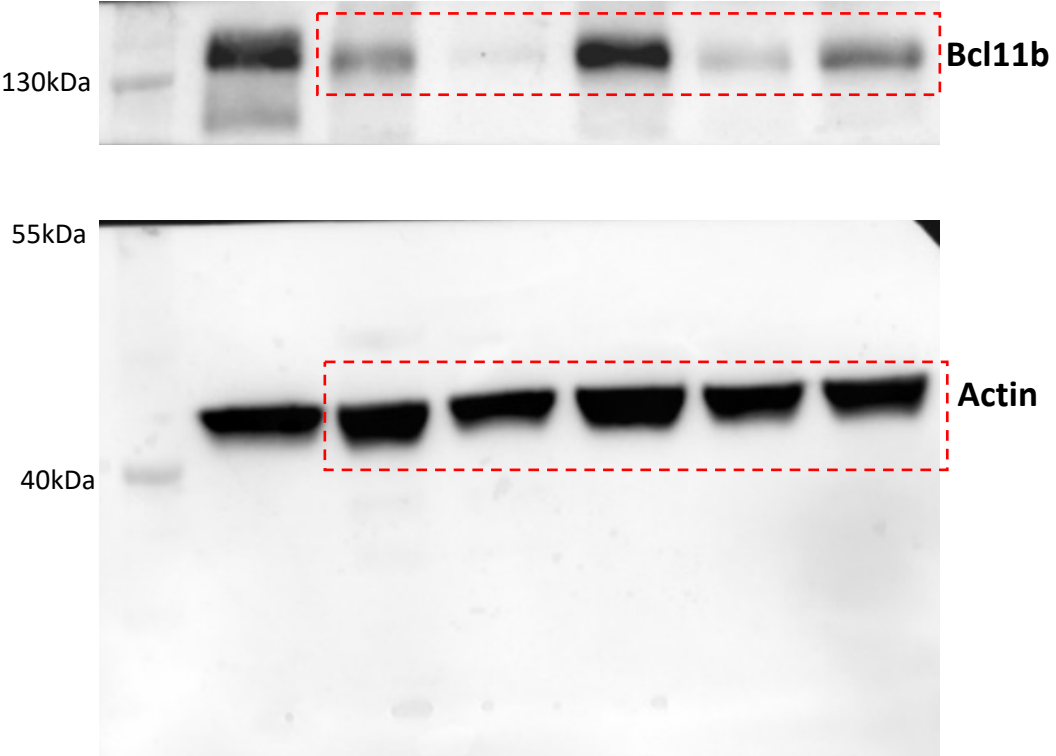

Figure 2t

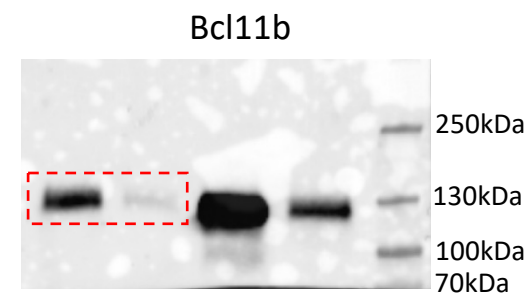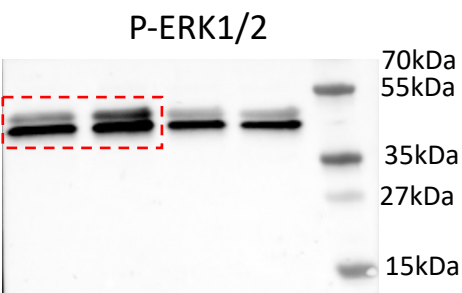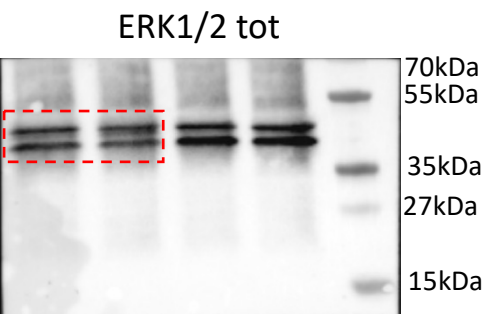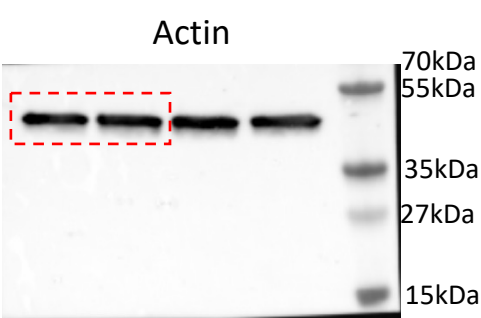

Supplement: Source Data Fig. 2 — Unprocessed western blots. [file 41556_2022_986_MOESM6_ESM.pdf]

Figure 4l

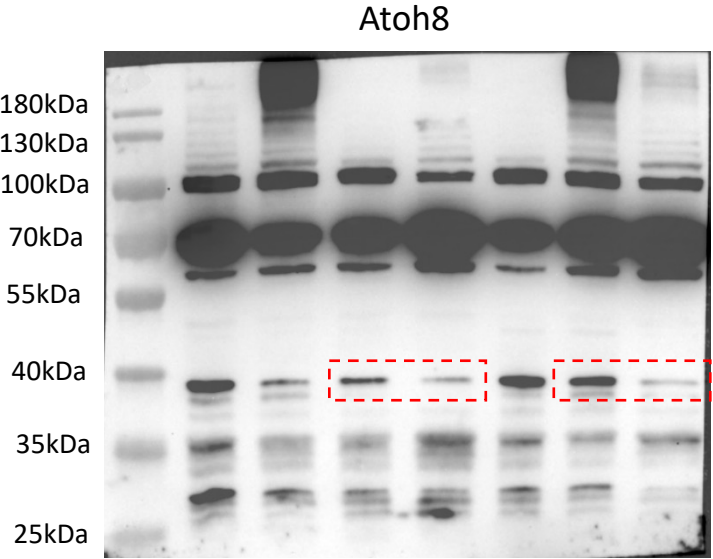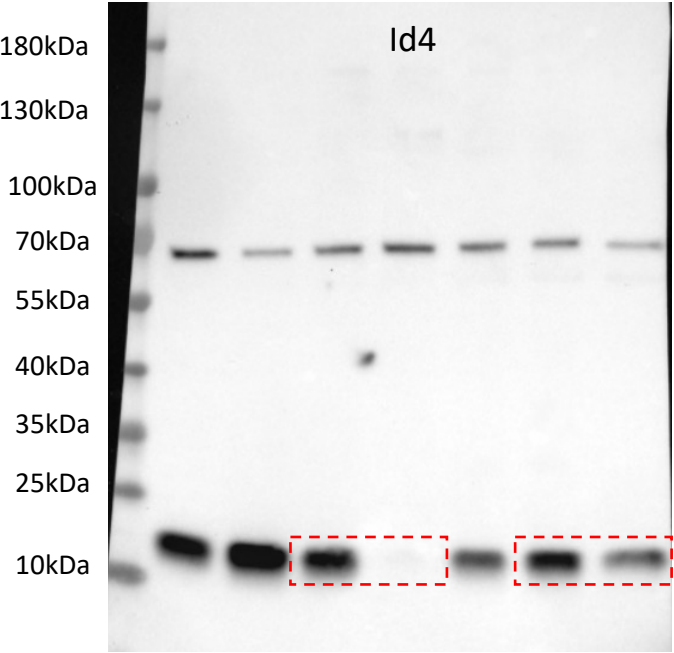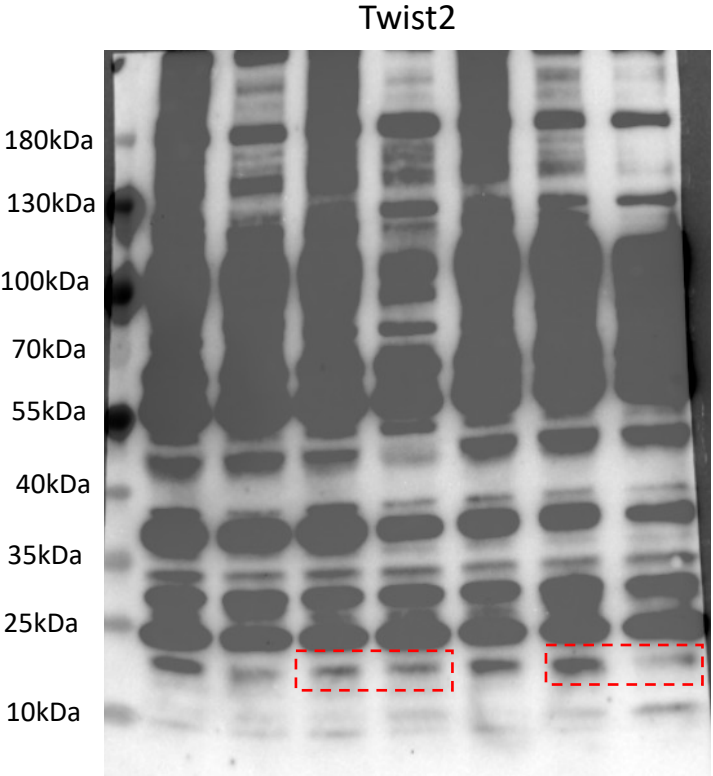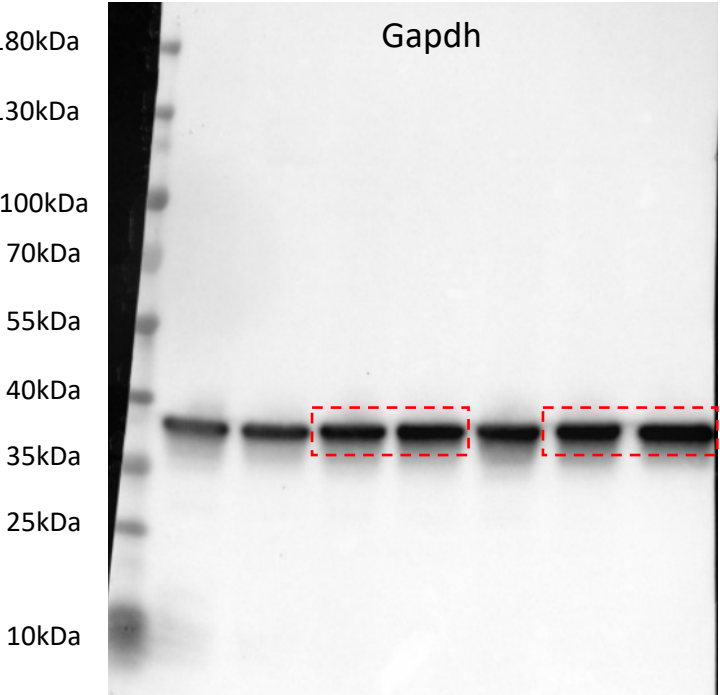

Figure 4m

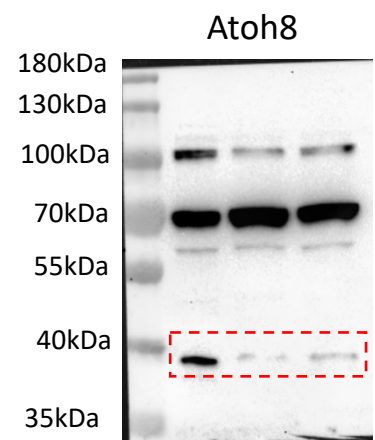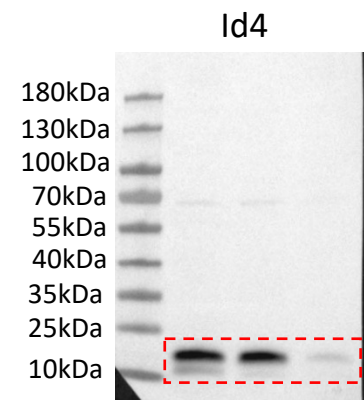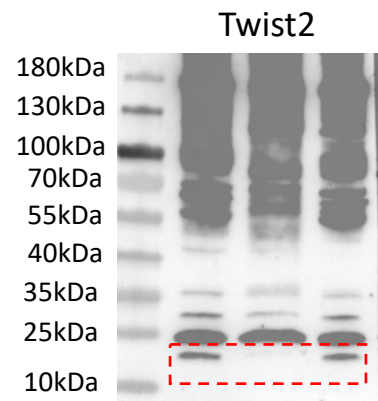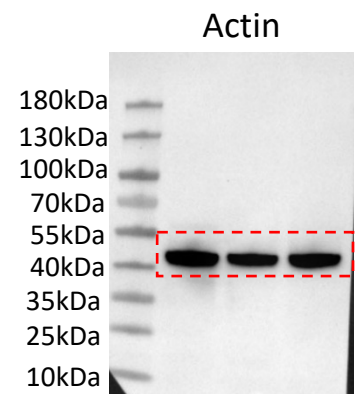

Supplement: Source Data Fig. 4 — Unprocessed western blots. [file 41556_2022_986_MOESM8_ESM.pdf]

Figure 5h

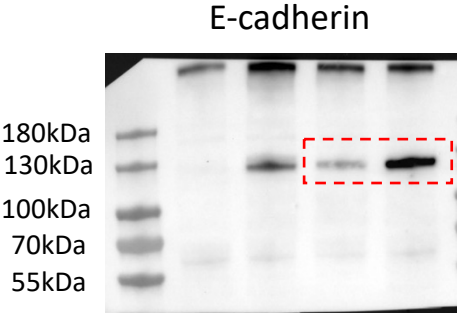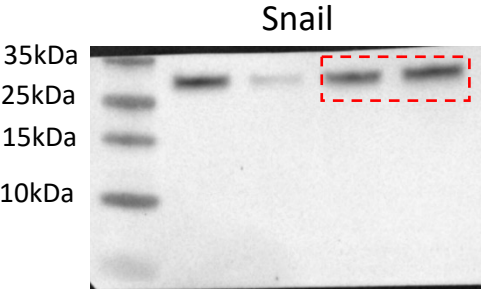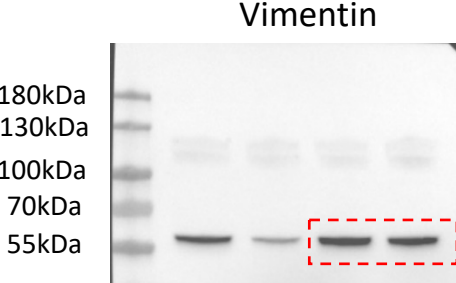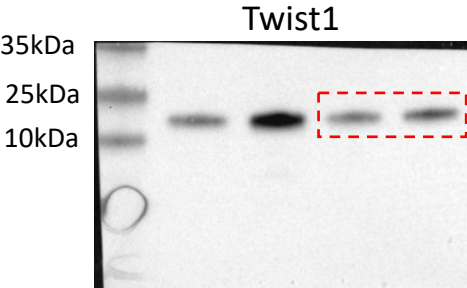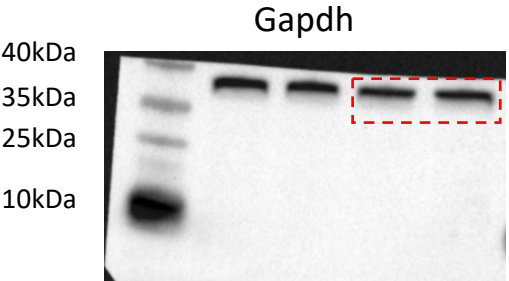

Supplement: Source Data Fig. 5 — Unprocessed western blots. [file 41556_2022_986_MOESM10_ESM.pdf]

Figure 6j

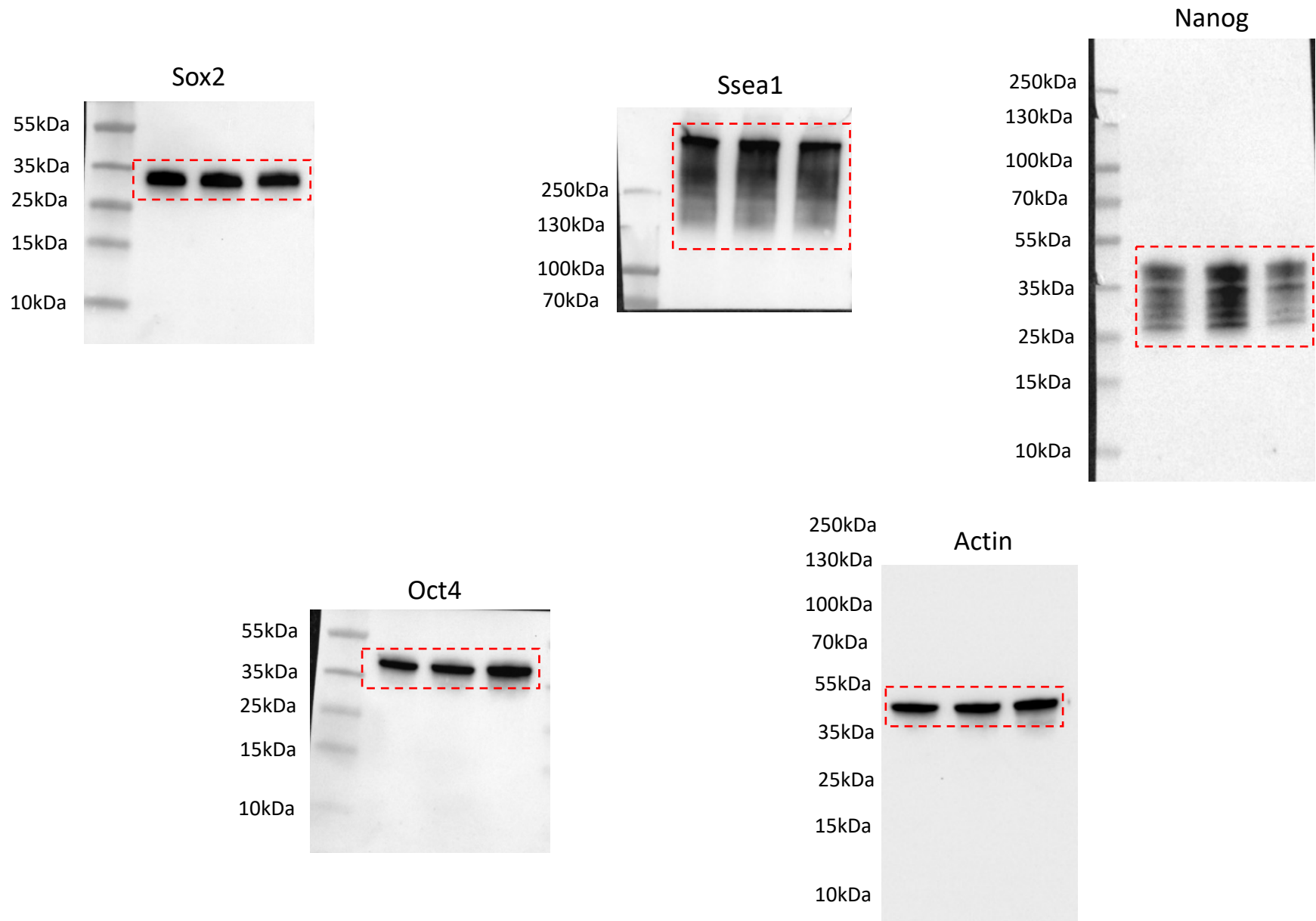

Supplement: Source Data Fig. 6 — Unprocessed western blots. [file 41556_2022_986_MOESM12_ESM.pdf]

Figure 7k

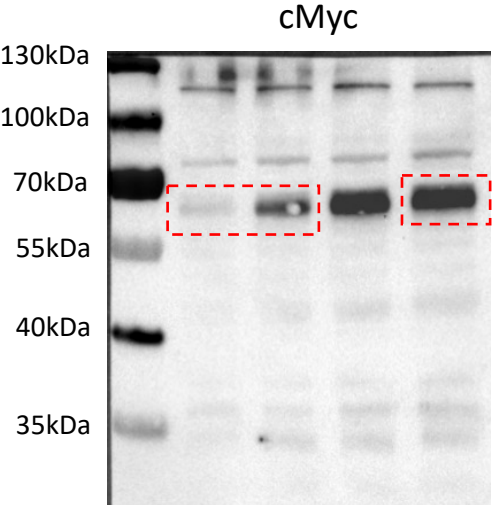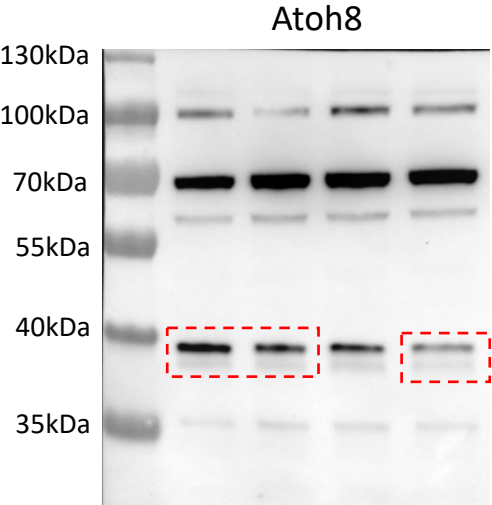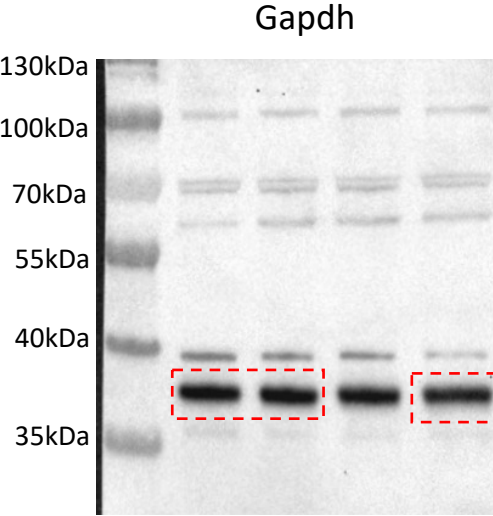

Figure 7r

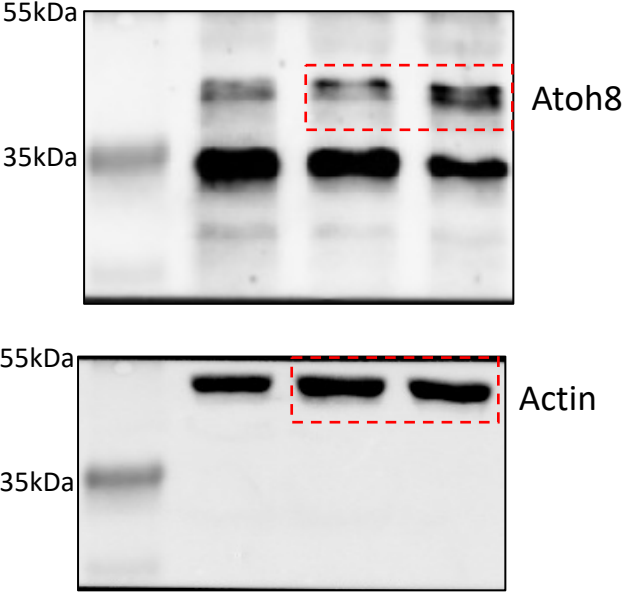

Supplement: Source Data Fig. 7 — Unprocessed western blots. [file 41556_2022_986_MOESM14_ESM.pdf]

Figure S2h

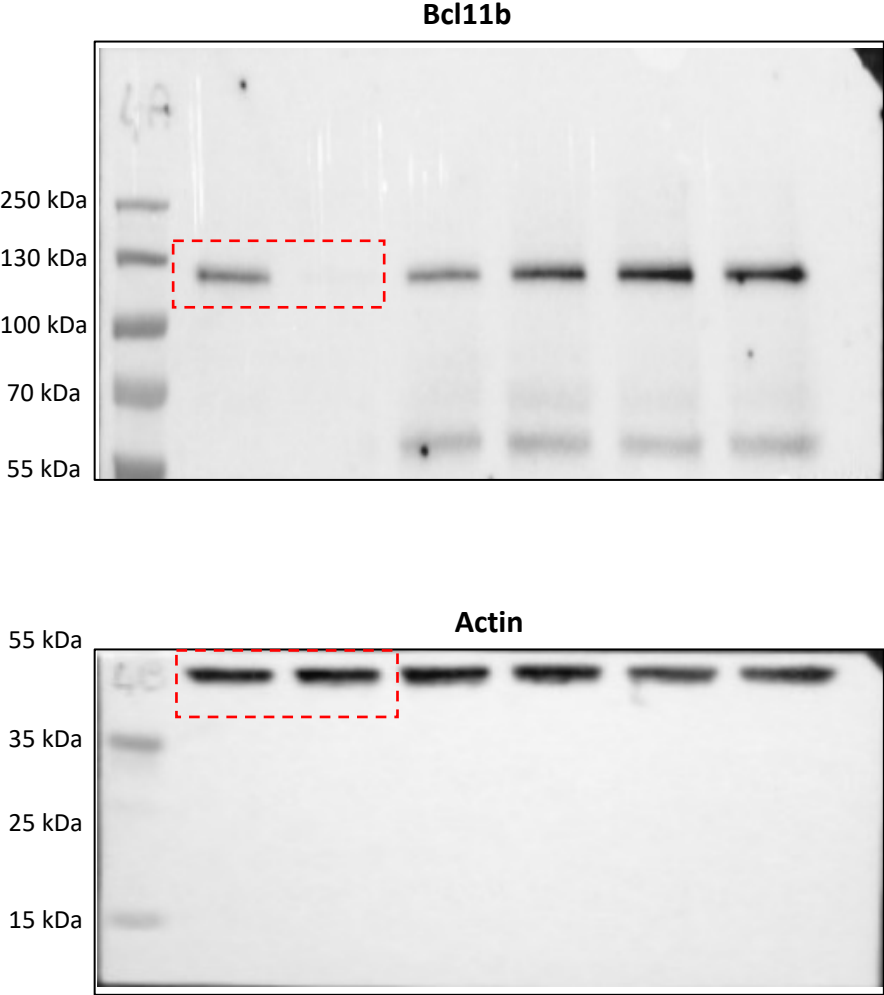

Figure S2n

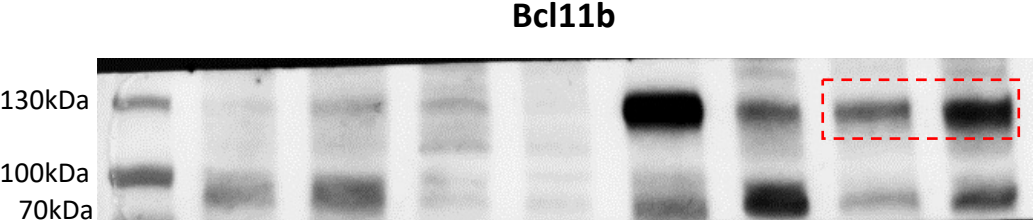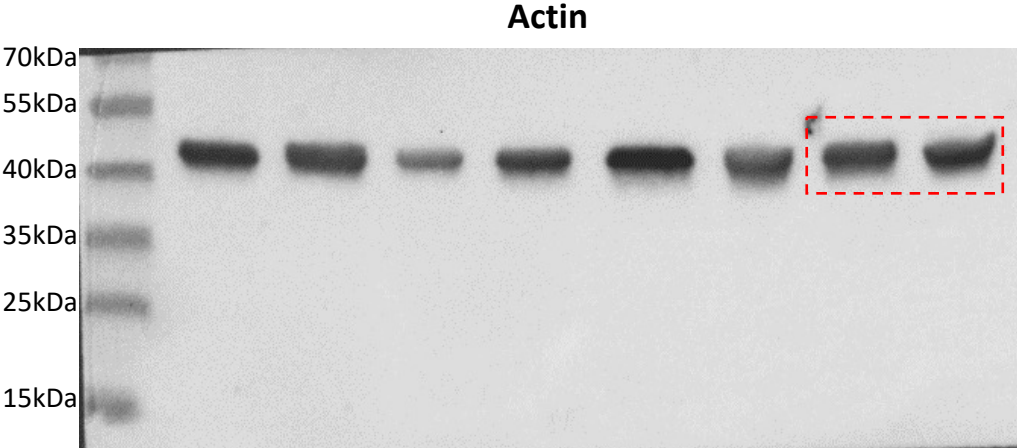

Supplement: Source Data Extended Data Fig. 2 — Unprocessed western blots. [file 41556_2022_986_MOESM17_ESM.pdf]

Figure S4e

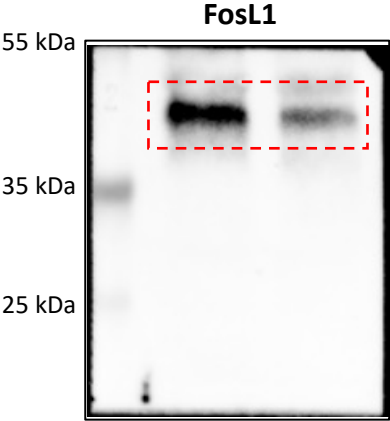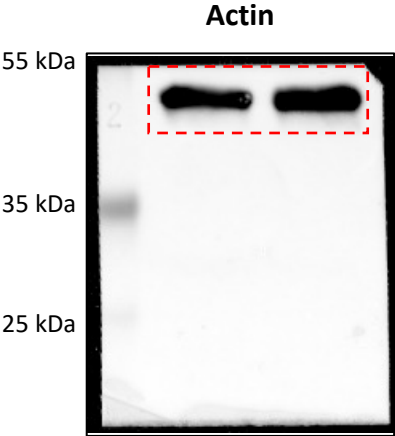

Supplement: Source Data Extended Data Fig. 4 — Unprocessed western blots. [file 41556_2022_986_MOESM20_ESM.pdf]

Figure S5l

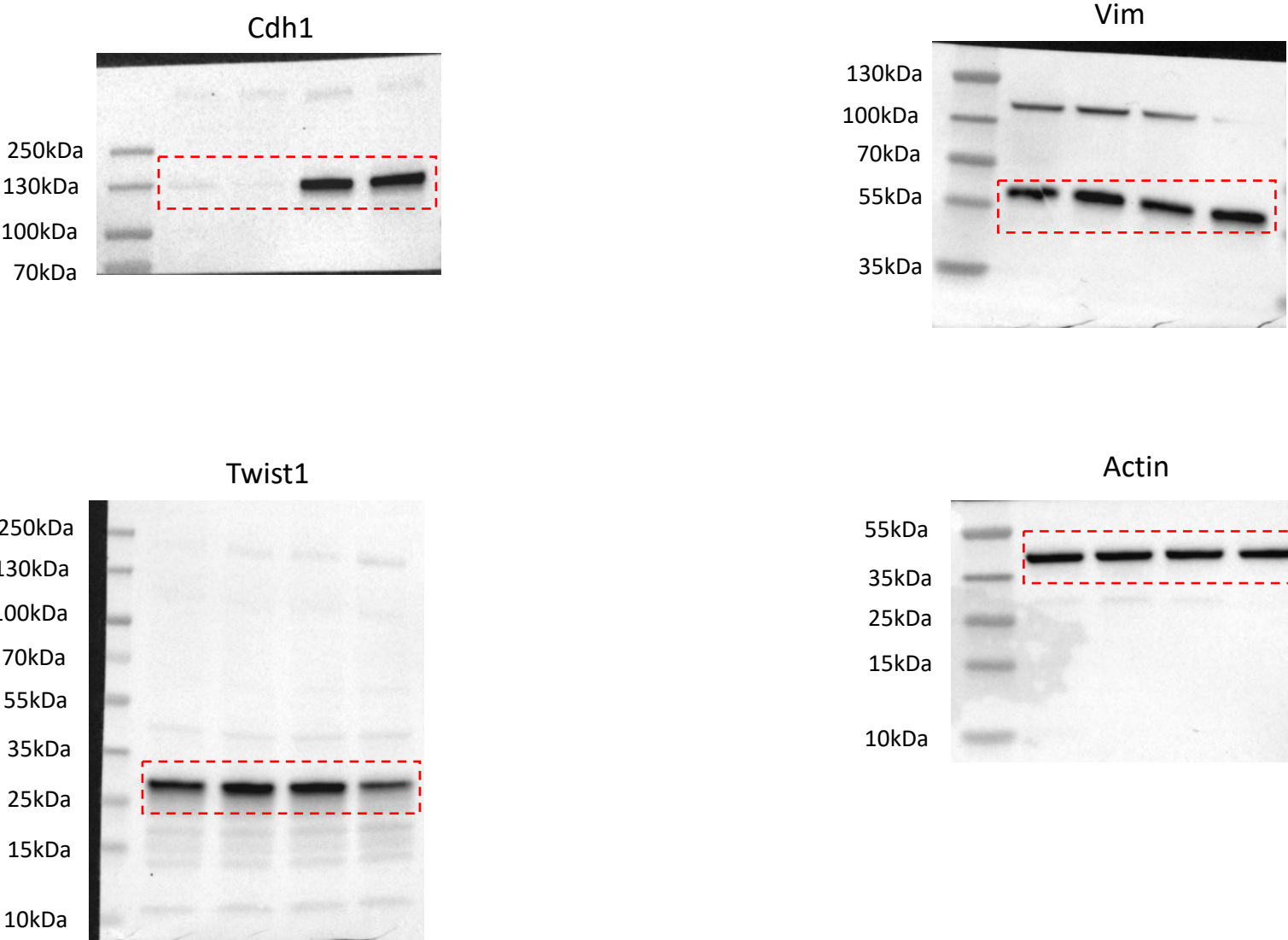

Supplement: Source Data Extended Data Fig. 5 — Unprocessed western blots. [file 41556_2022_986_MOESM22_ESM.pdf]

Figure S6b

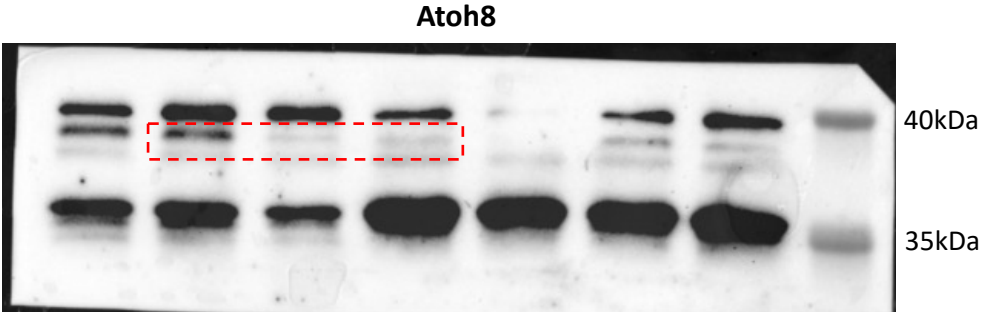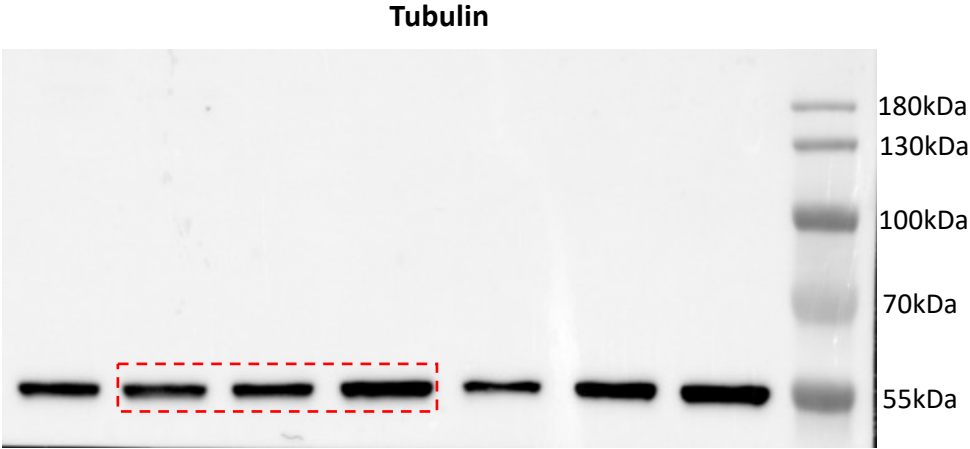

Figure S6d

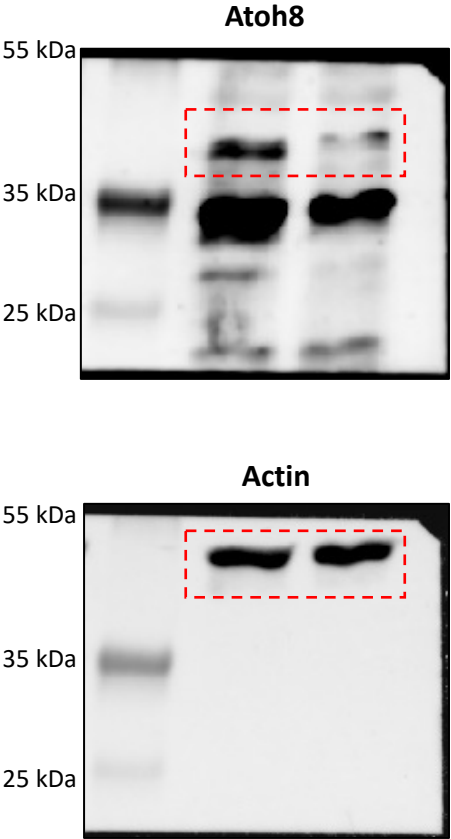

Figure S6h

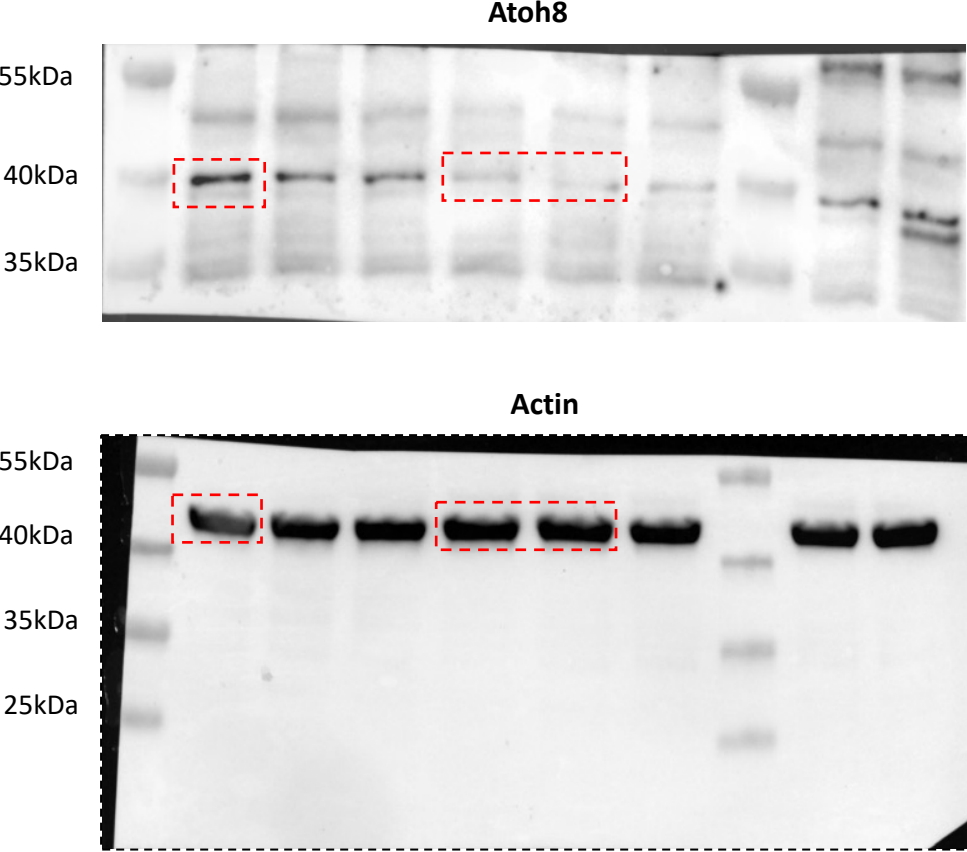

Figure S6k

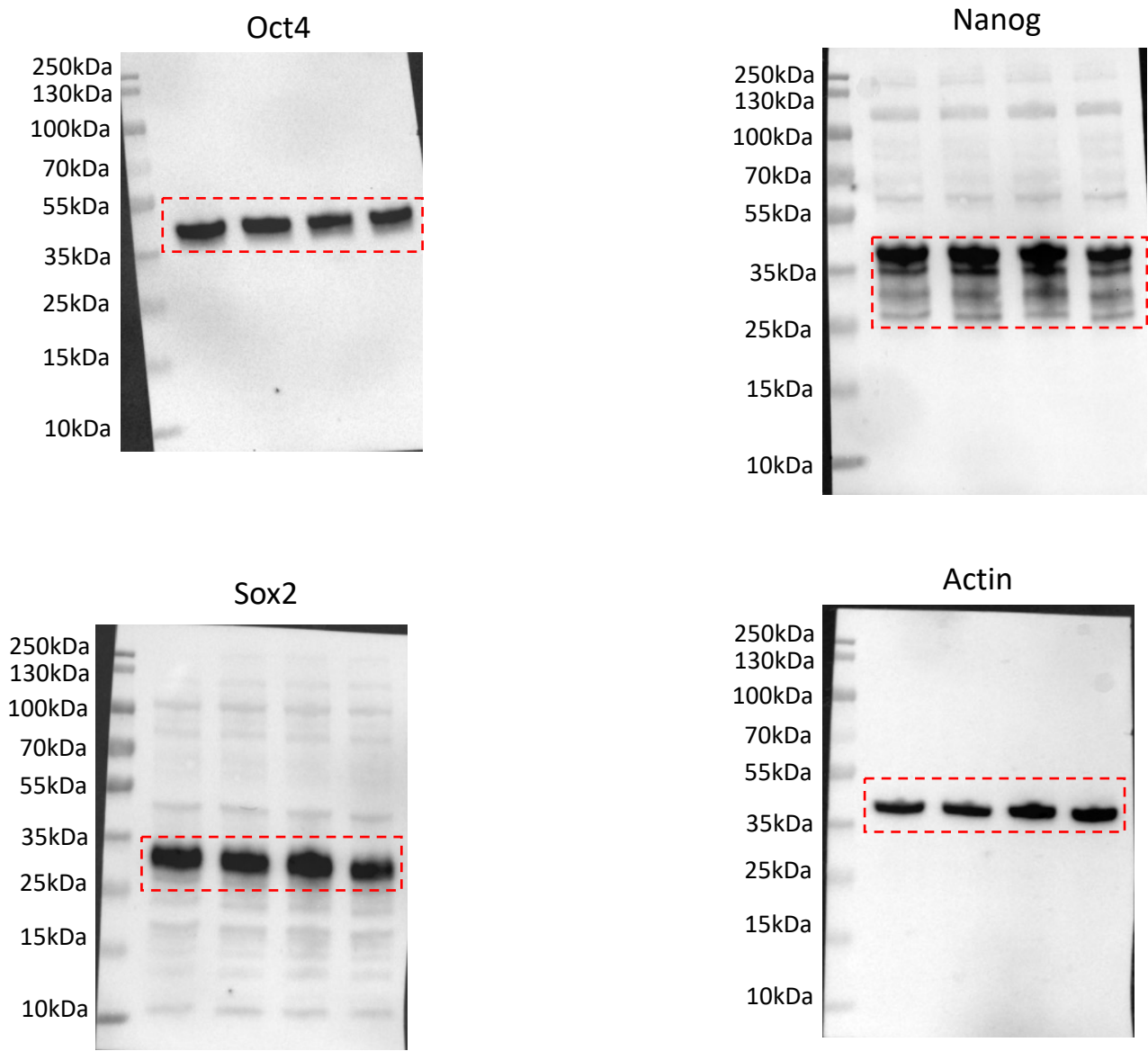

Supplement: Source Data Extended Data Fig. 6 — Unprocessed western blots. [file 41556_2022_986_MOESM24_ESM.pdf]

Figure S7d

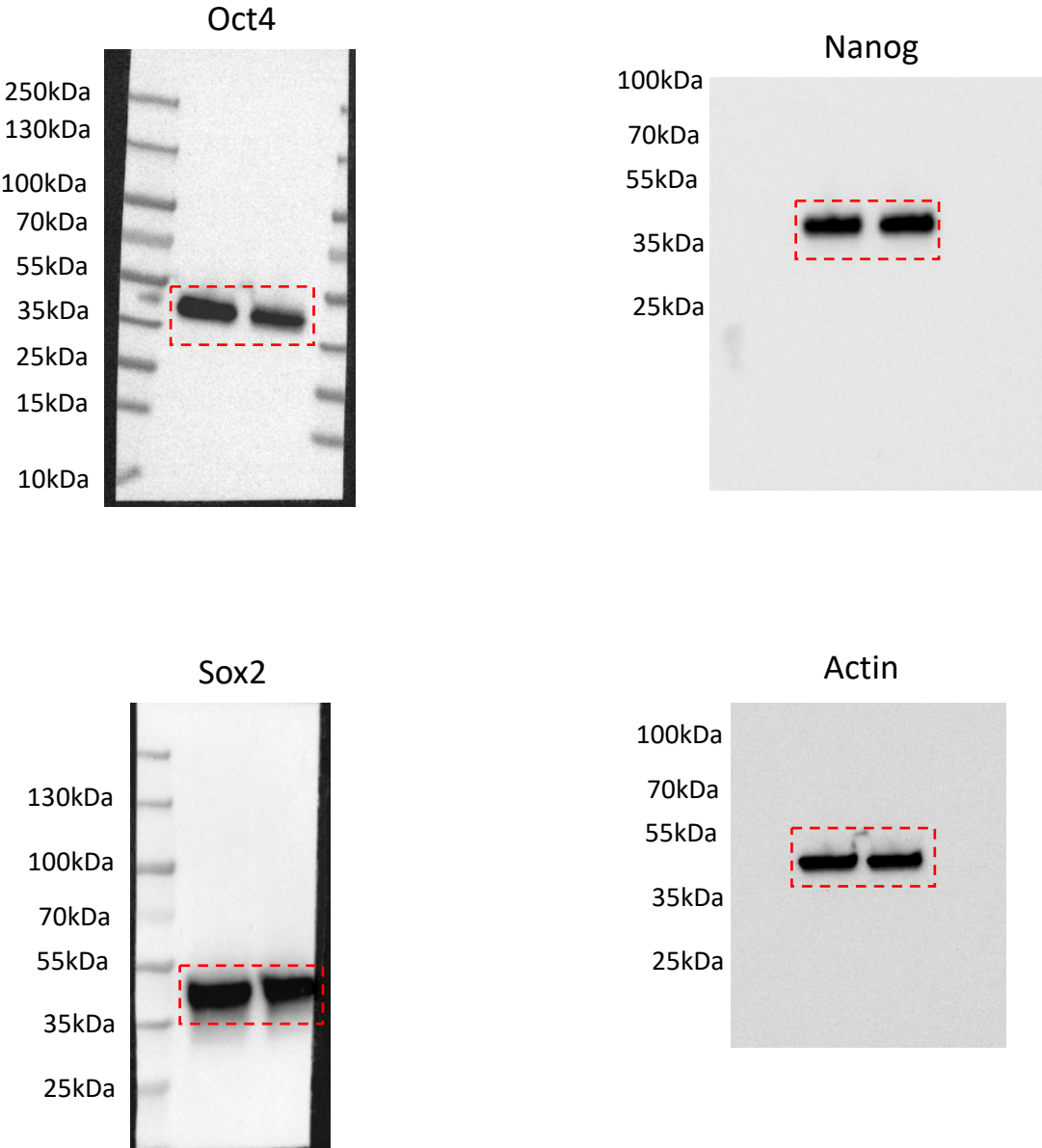

Figure S7f

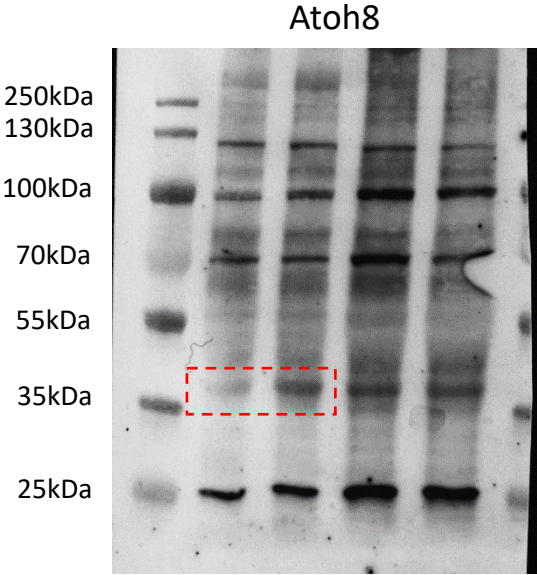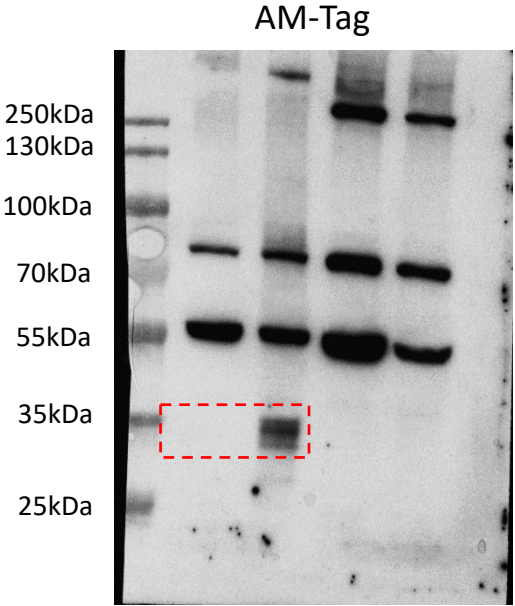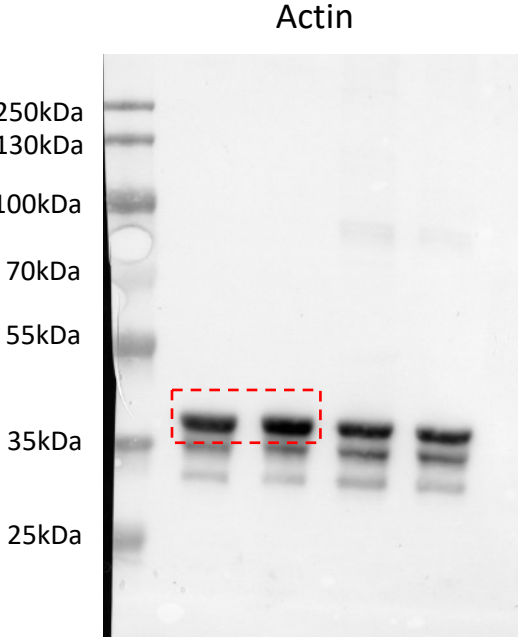

Figure S7m

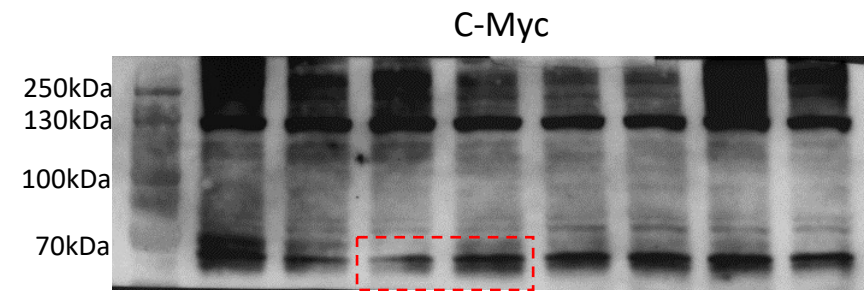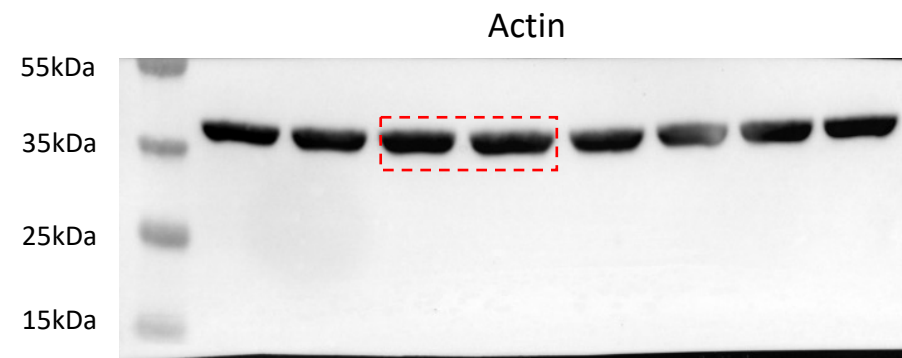

Figure S7n

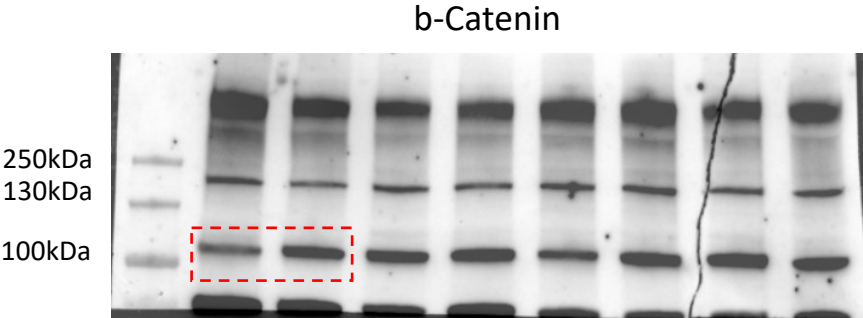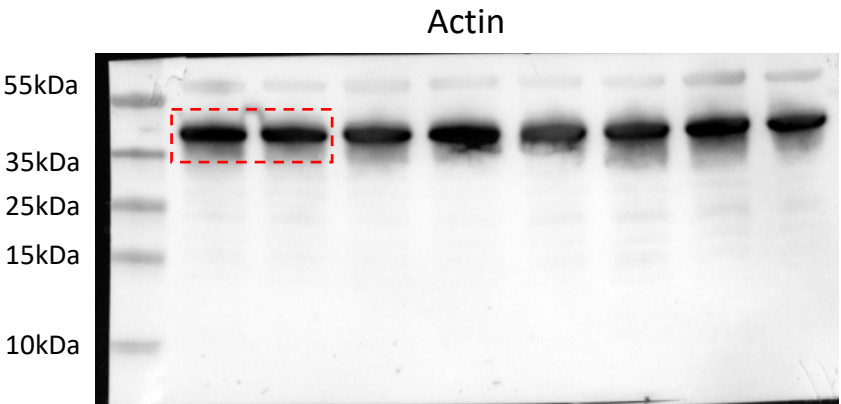

Figure S7q

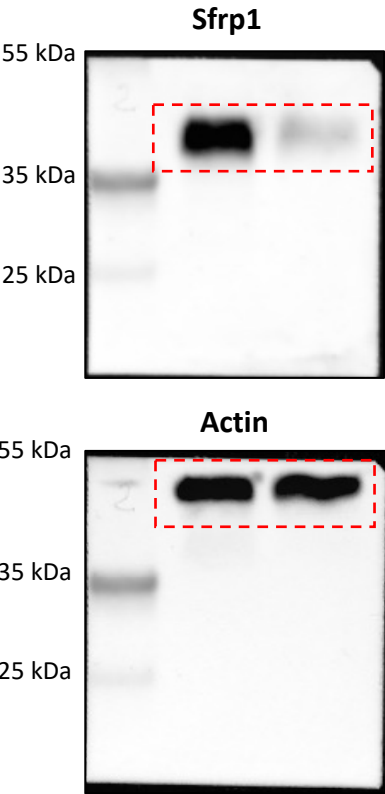

Figure S7r

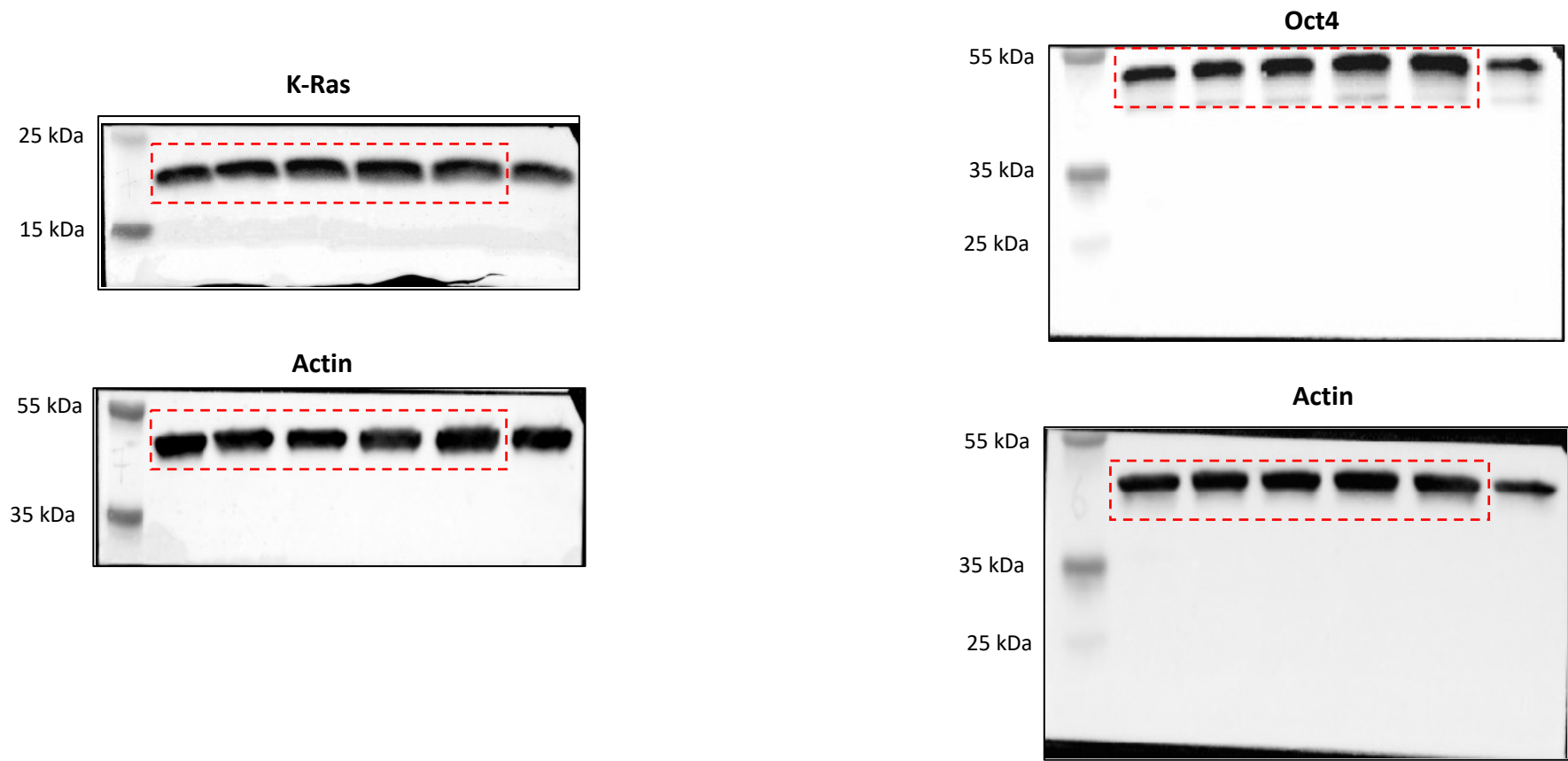

Figure S7s

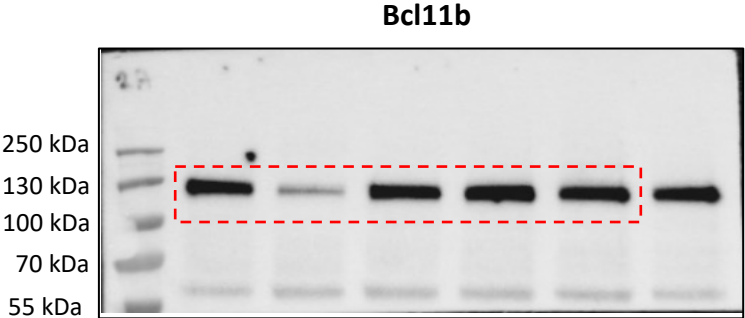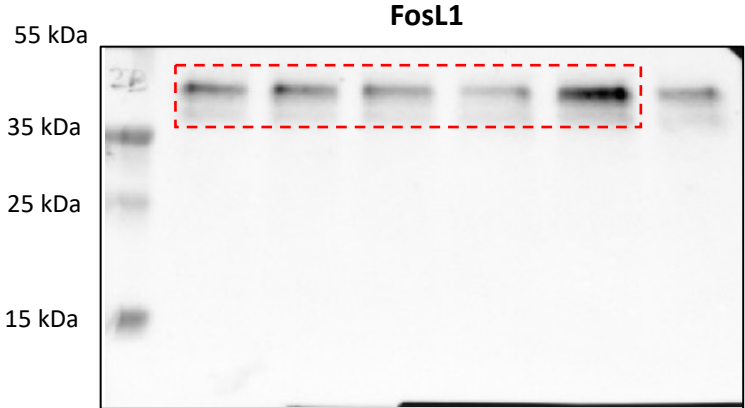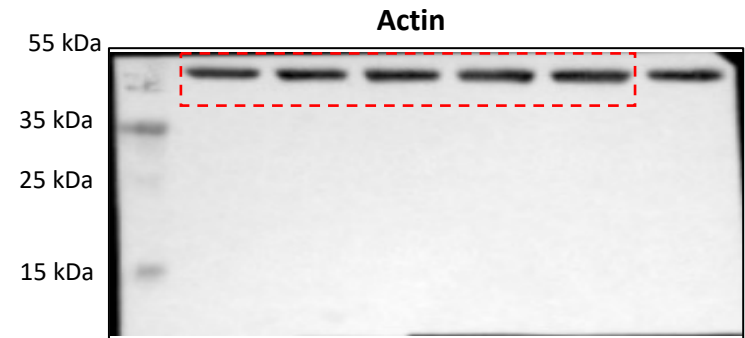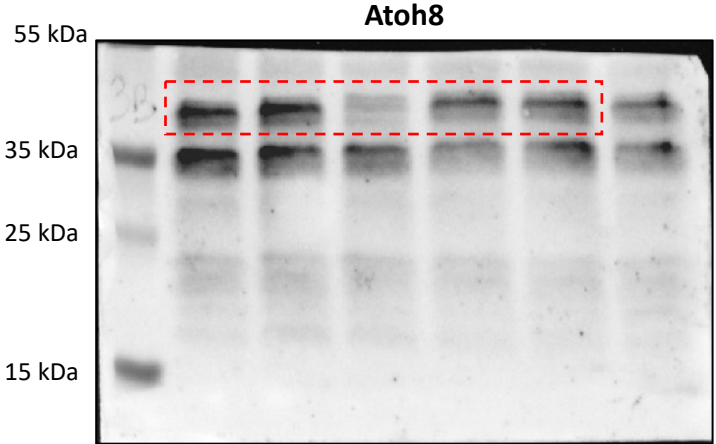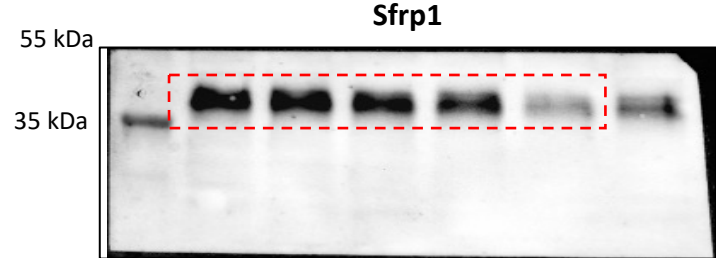

Supplement: Source Data Extended Data Fig. 7 — Unprocessed western blots. [file 41556_2022_986_MOESM26_ESM.pdf]
